# Soil-transmitted helminth infections and leprosy: a cross-sectional study of the association between two major neglected tropical diseases in Indonesia

**DOI:** 10.1186/s12879-016-1593-0

**Published:** 2016-06-08

**Authors:** Salma Oktaria, Evita Halim Effendi, Wresti Indriatmi, Colette L. M. van Hees, Hok Bing Thio, Emmy Soedarmi Sjamsoe-Daili

**Affiliations:** Department of Dermatology and Venereology, Faculty of Medicine, Universitas Indonesia, Jakarta, Indonesia; Department of Dermatology, Erasmus University Medical Center, Rotterdam, The Netherlands

**Keywords:** Helminth coinfection, Type of leprosy, Type 2 leprosy reaction

## Abstract

**Background:**

The clinical spectrum of leprosy is dependent on the host immune response against *Mycobacterium leprae* or the newly discovered *Mycobacterium lepromatosis* antigen. Helminth infections have been shown to affect the development of several diseases through immune regulation and thus may play a role in the clinical manifestations of leprosy and leprosy reactions. The purpose of this study is to determine the proportion of helminth infections in leprosy and its association with the type of leprosy and type 2 leprosy reaction (T2R).

**Methods:**

History or episode of T2R was obtained and direct smear, formalin-ether sedimentation technique, and Kato-Katz smear were performed on 20 paucibacillary (PB) and 61 multibacillary (MB) leprosy participants.

**Results:**

There are more helminth-positive participants in MB leprosy compared to PB (11/61 versus 0/20, *p* = 0.034) and in T2R participants compared to non-T2R (8/31 versus 3/50, *p* = 0.018).

**Conclusions:**

Our results suggest that soil-transmitted helminth infections may have a role in the progression to a more severe type of leprosy, as well as the occurrence of T2R. These findings could serve as a fundamental base for clinicians to perform parasitological feces examination in patients who have MB leprosy and severe recurrent reactions to rule out the possibility of helminth infection. Further secondary confirmation of findings are needed to support these conclusions.

## Background

Leprosy is a chronic granulomatous infectious disease caused by an obligatory intracellular pathogen *Mycobacterium leprae* or the newly discovered *Mycobacterium lepromatosis*. These organisms are known to have a unique high affinity for Schwann cells (neurotropism), but it can affect most human organs with the exception of the central nervous system. Depend on the host immune response, the clinical manifestations of leprosy may range from minor skin lesions, nerve damage, to deformities and systemic involvement [1, 2].

Leprosy is one of the oldest known human diseases yet still one of the major infectious diseases in the world, particularly in developing countries. Globally, new case detection rates and registered prevalence rates of leprosy have remained stable over the last decade, indicating stagnation in leprosy control. Despite the successful implementation of multidrug therapy, there were still 14 countries that reported more than 1000 new cases of leprosy in 2013, including Indonesia. The Ministry of Health Republic of Indonesia reported that there were 16,856 new cases in 2013, with a registered prevalence around 19,730 cases. The majority of new cases (14,062 cases) were multibacillary (MB) leprosy and 1694 cases presented with grade 2 disabilities [3].

Immunologic reaction is one important factor that may impact the course of the disease as well as the occurrence of associated disabilities. Leprosy reactions may occur in 30–50 % of patients and can happen any time during the course of leprosy. Type 2 reaction (erythema nodosum leprosum [T2R]) is the most frequent reaction found in the multibacillary form of leprosy and is often associated with bacterial and parasite infections [4, 5].

As leprosy primarily affects the poorest population living in the remote or rural areas, helminth infection is not uncommonly found as a co-morbidity in leprosy. Systemic corticosteroids are the first-line drugs used for treating leprosy reactions. However, their long-term and repeated usage may affect host immune system and cause a condition that helps maintain helminth infection in a vicious cycle. For this reason, fecal examination for the presence of helminth ova or larvae and antihelminthic therapy prior to corticosteroid therapy in all leprosy patients has been recommended by the International Federation of Anti-Leprosy Association (ILEP) [6]. However, studies regarding the association of helminth infection with the clinical spectrums of leprosy are still limited thus the recommendation has not been applied in daily clinical practice in Indonesia.

In addition to triggering T2R, helminth infection has also been demonstrated to alter the host immune response and thus may have a role in the course of several diseases [7]. Taking into consideration that the clinical spectrums of leprosy represents the host immune response against *M. leprae* or *Mycobacterium lepromatosis*, helminth co-infection may also play a role in determining the clinical manifestations of leprosy by altering the host immune response against *M. leprae* or *M. lepromatosis* antigen. The purpose of this study is to determine the proportion of helminth infections in Indonesian adult population affected by leprosy and its association with the type of leprosy and T2R. This is the first study in Indonesia focusing on the two major neglected tropical diseases in Indonesia, helminth infections and leprosy.

## Methods

### Study sites and participants

This study was conducted in Cipto Mangunkusumo Hospital, Jakarta and Dr. Sitanala Hospital, Tangerang from October 2013 to April 2014. The study was approved by the medical ethical committee of the Faculty of Medicine, Universitas Indonesia. Inclusion criteria were leprosy patients aged 18–60 years old, starting 4 months of WHO multidrugs therapy (MDT) until one year release from treatment, and agreement to sign an informed consent. Leprosy patients who consume MDT other than the WHO regimen, have a history of antihelminthic therapy three months prior to the study, a history of laxative and hypertonic saline consumption 14 days prior to the study, and were known to have severe systemic diseases, tuberculosis or HIV-AIDS, were excluded from the study. Twenty PB leprosy and 61 MB leprosy patients were enrolled after they signed the informed consent.

### Field and laboratory procedures

All participants were evaluated according to anamnesis, clinical examination, and slit skin smear examination, as well as secondary data from medical records. Participants were classified according to both WHO and Ridley-Jopling criteria. Type 2 leprosy reaction was defined as a sudden eruption of painful erythematous papules, nodules, or plaques that may be ulcerated and/or accompanied by fever, malaise, peripheral edema, arthralgia/arthritis, nerve impairments, eye involvement, lymphadenitis, or epididimo-orchitis. Based on their slit skin smear results and the presence of T2R, participants were grouped into two groups of PB (negative slit skin smear results, including indeterminate and tuberculoid leprosy) and MB leprosy (positive slit skin smear results, including borderline and lepromatous leprosy), as well as two groups of T2R and no T2R.

For the assessment of soil-transmitted helminth infection, minimum one gram of self-collected fecal sample was obtained from all participants and analyzed by experienced laboratory technicians using direct smear and formalin-ether sedimentation technique based on the WHO recommendation for the presence of helminth ova or larvae [8]. Diagnosis of helminth infection was made if a minimum of one ovum or larva was found in the fecal sample. In addition, a single Kato-Katz smear was also performed to determine the intensity of helminth infections according to WHO guidelines as light, moderate, and heavy-intensity infection [9]. Based on the parasitological examination results, participants were divided into another two groups of helminth-positive and helminth-negative participants. Antihelminthic therapy was given to helminth-positive participants according to the standard operational procedure.

### Statistical analysis

Data were collected and recorded in the clinical research form. Microsoft Excel 2010 and Stata version 12 data analysis and statistical software of StataCorp. USA were used for editing, coding, data entry, and data analysis. The Fischer exact test was used to determine the proportion of helminth infections and its association with the type of leprosy and T2R. Statistical significance was defined as *p* < 0.05.

## Results

### Participants’ demographics and clinical features

Eighty one participants that met the eligibility criteria were enrolled in this study; with a male to female ratio of 4.8:1. The age ranged from 20 to 58 years (mean 33.47 years, standard deviation 9.22). The majority of participants were aged between 30–44 years old (53.1 %) and of mid-educational level (55.6 %), followed by a low educational level (35.8 %). Demographic features of the study participants are summarized in Table 1.

**Table 1:** Demographic features of study participants between October 2013 and April 2014 (*n* = 81)

No	Demographic features	Leprosy type n (%)		Total n (%)
		Paucibacillary (*n* = 20)	Multibacillary (*n* = 61)	
1	Gender			
	• Male	13 (65.0)	54 (88.5)	67 (82.7)
	• Female	7 (35.0)	7 (11.5)	14 (17.3)
2	Age groups			
	• 18–29 years	6 (30.0)	22 (36.1)	28 (34.6)
	• 30–44 years	9 (45.0)	34 (55.7)	43 (53.1)
	• 45–60 years	5 (25.0)	5 (8.2)	10 (12.3)
3	Educational level			
	• Low	6 (30.0)	23 (37.7)	29 (35.8)
	• Middle	11 (55.0)	34 (55.7)	45 (55.6)
	• High	3 (15.0)	4 (6.6)	7 (8.6)

Most of the participants (60.5 %) were diagnosed with borderline lepromatous leprosy and 75.3 % of all patients had MB leprosy with positive smears. Approximately 75.3 % participants were on MDT at inclusion and 31 participants (38.3 %) had a history of or episode of T2R. Among the participants with T2R, 17 participants (54.8 %) had T2R during MDT consumption and 17 participants (54.8 %) received systemic corticosteroid therapy for more than 12 weeks for one T2R episode. Clinical features of the study participants are summarized in Table 2.

**Table 2:** Clinical features of study participants between October 2013 and April 2014 (*n* = 81)

No.	Clinical features	n	Percentage (%)
1.	Leprosy type (Ridley & Jopling)		
	• Indeterminate	0	0
	• TT	1	1.2
	• BT	19	23.5
	• BB	2	2.5
	• BL	49	60.5
	• LL	10	12.3
2.	Leprosy type		
	• PB	20	24.7
	• MB	61	75.3
3.	Status of medication		
	• On fourth months of MDT-WHO until completion of treatment	61	75.3
	• RFT ≤ 6 months	11	13.6
	• RFT > 6 months-1 year	9	11.1
4.	History of T2R		
	• No	50	61.7
	• Yes	31	38.3
5.	Onset of T2R		
	• Before MDT-WHO treatment	8	25.8
	• During MDT-WHO treatment	17	54.8
	• After completion of MDT-WHO treatment	6	19.4
6.	Duration of corticosteroid therapy for T2R		
	• ≤ 12 weeks	14	45.2
	• > 12 weeks	17	54.8

### Assessment of helminth infections

Helminth infections were found in 11 of 81 participants (13.6 %). Five participants (6.2 %) were having light infections of *Trichuris trichiura* with 1–8 eggs/g of fecal samples, while 6 participants (7.4 %) were having heavy *Strongyloides stercoralis* infections with a large quantities of *S. stercoralis* larvae by either direct microscopic examination, formalin-ether sedimentation technique, or Kato-Katz smear. The clinical features of 11 helminth-positive participants and the association of helminth infection with the type of leprosy and T2R are consecutively summarized in Tables 3 and 4. Among the 11 participants (13.6 %) that had helminth infection, all belonged to the smear-positive MB group and 8 of them had a history of or were experiencing T2R. Based on statistical analyses, it can be concluded that there were significant associations of helminth infection with the type of leprosy (*p* = 0.034) and T2R (*p* = 0.018).

**Table 3:** Clinical features of helminth-positive participants (*n* = 11)

Participant number	Type of leprosy		Parasites	Intensity of infection
	Ridley-Jopling	WHO		
5	BL	MB	*Trichuris trichiura*	Mild
9	BL	MB	*Trichuris trichiura*	Mild
19	BL	MB	*Trichuris trichiura*	Mild
31	BL	MB	*Strongyloides stercoralis*	Severe
33	LL	MB	*Trichuris trichiura*	Mild
47	BL	MB	*Strongyloides stercoralis*	Severe
48	LL	MB	*Strongyloides stercoralis*	Severe
49	LL	MB	*Trichuris trichiura*	Mild
50	BL	MB	*Strongyloides stercoralis*	Severe
55	BL	MB	*Strongyloides stercoralis*	Severe
66	BL	MB	*Strongyloides stercoralis*	Severe

Note. *BL* bordeline lepromatous, *LL* lepromatous leprosy

**Table 4:** Helminthiasis in participants and its association with leprosy type and type 2 reaction (*n* = 81)

	Helminth-negative n (%)	Helminth-positive n (%)	Total n (%)	*p* value
Paucibacillary	20 (100)	0 (0)	20 (24.7)	0.034
Multibacillary	50 (82)	11 (18)	61 (75.3)	
Total	70 (86.4)	11 (13.6)	81 (100)	
No T2R	47 (94)	3 (6)	50 (61.7)	0.018
T2R	23 (74.2)	8 (25.8)	31 (38.3)	
Total	70 (86.4)	11 (13.6)	81 (100)	

## Discussion

The global data of 2010 stated that the helminth-infected population in South-East Asia is caused mainly by *Ascaris lumbricoides* (126.7 million people), followed by *T. trichiura* (115.3 million people) [10]. In this study, we only observed 11 smear-positive MB leprosy participants (13.6 %) who are co-infected by either *T. trichiura* or *S. stercoralis*. The fact that we did not find *A. lumbricoides* in the participants might be explained by varied prevalence rates of soil-transmitted helminth infection (STH) between districts in Indonesia. Furthermore, the data regarding helminthic infection status in Indonesian adult population is still scarce as most of the studies were conducted in pre-school and school age population [11, 12].

In addition to varied prevalence of STH between districts and age groups, the number of helminth infection in this study may also be underestimated due to an uneven distribution in the daily excretion of small number of ova, particularly in mild infection. Likewise, the sample collecting procedures could also have influenced the results of the examination. For example, the result may not be positive if the collected part of the stool does not contain helminth ova . Multiple and serial collection of fecal samples are expected to increase the positivity of helminth infection.

Ruling out the possibility of helminth infection before and during corticosteroid therapy is particularly relevant considering that long-term systemic corticosteroid use for treating leprosy reactions may predispose to a new helminth infection or aggravate the pre-existing infection. Generalized serpiginous eruption and death caused by *S. stercoralis* hyperinfection during immunosuppressive treatment for leprosy reaction have been reported in Brazil and Cambodia [13, 14]. In this study, severe *S. stercoralis* was observed in 6 participants who had a history or were experiencing T2R and undergoing long-term corticosteroid treatment at inclusion. Most of T2R will become better in 2 weeks even without therapy. However, the expected outcome was not observed in most of T2R participants who are also helminth-positive. Most of them are consuming systemic corticosteroid therapy for more than 12 weeks due to alternate clinical deterioration following an improvement. Although it was not specifically studied, a clinical improvement of T2R was then observed in helminth-positive participants two weeks after albendazole therapy 400 mg daily for 3 consecutive days.

Regarding the immunomodulatory properties of helminth infection, it has been demonstrated that certain helminth-derived proteins can skew the host immune response towards Th2. Taking into account that the effectiveness of the immune response against mycobacterial infection depends on the Th1/Th17 response, it is possible that helminth co-infection may facilitate *M. leprae* or *M. lepromatosis* growth and dissemination through the upregulation of Th2 cytokines or CD4 + CD25+ regulatory T cells (Tregs) [15, 16]. Previous studies indicated that the presence of intestinal helminth infections may have a role in facilitating *M. leprae* infection or the progression to more disseminated and MB forms of leprosy. Prost and colleagues [17] reported that of individuals that live in two different geographical areas with the same prevalence of leprosy cases, those living in onchocerciasis hyperendemic areas had a higher prevalence of MB leprosy. Furthermore, Diniz and colleagues [18] reported the decrease of interferon-γ and the increase of Th2 cytokines IL-4 and IL-10 levels in lepromatous leprosy patients co-infected with STH.

As helminth infection induces a strong Th2 immune response, it may also exacerbate T2R. In this study, history or episode of T2R was found in 31 participants (38.3 %). Eight of T2R participants were found to have STH infection, which is significantly associated with the occurrence of T2R. However, it is still not clear how STH could contribute to the occurrence of T2R. The role of STH on the course of leprosy may not only be a direct process but may occur by influencing other factors associated with leprosy, which is may also be the reason why there were 3 helminth-positive participants in this study who were not developing T2R.

Despite the low positivity rate of STH in this study, a significant association between helminth infection with MB leprosy and T2R should not be neglected. Additionally, several other potential factors have also been described in regards to the development of leprosy, including genetics [19], age [20], gender [21], as well as contact duration and distance [22]. These factors may influence the development of leprosy in a synergic manner. However, the clear mechanism of how intestinal helminth may contribute to the increased prevalence of MB leprosy and T2R, as well as the interplay mechanisms between helminth and other risk factors for leprosy remains poorly understood. This is a pilot study that was conducted due to lack of scientific data regarding intestinal helminth infections and leprosy in Indonesia. Larger and more advanced research is currently being conducted to elucidate the role of other factors in regards to the presence of intestinal helminth infections in leprosy at the molecular level.

## Conclusions

Our results suggest that soil-transmitted helminth infections may have a role in the progression to a more severe type of leprosy, as well as the occurrence of T2R. These findings could serve as a fundamental base for clinicians to perform parasitological feces examination in patients who have MB leprosy and severe recurrent reactions to rule out the possibility of helminth infection. This is particularly relevant in those on or with a history of long-term corticosteroid treatment. However, further studies are required to investigate how intestinal helminths could contribute to increased prevalence of MB leprosy and T2R. Larger and more advanced research is currently being conducted to elucidate the role of other factors in regards to the presence of intestinal helminth infections in leprosy at the molecular level.

## Abbreviations

HIV-AIDS, human immunodeficiency virus-acquired immunodeficiency syndrome; IL, interleukin; MB, multibacillar; MDT, multidrug therapy; PB, paucibacillar; STH, soil-transmitted helminth; T1R, type 1 leprosy reaction; T2R, type 2 leprosy reaction; TGF-β, tumour growth factor-β; Th, T-helper; WHO, World Health Organization
